# Myocardial Stunning Induced by Vasospasm Within an Intercoronary Communication

**DOI:** 10.1016/j.jaccas.2026.107281

**Published:** 2026-03-10

**Authors:** Genryu Mizokami, Shunsuke Katsuki, Yasuhiro Nakano, Keiji Oi, Toyokazu Uwatoku, Tetsuya Matoba, Hiroyuki Tsutsui, Kohtaro Abe

**Affiliations:** Faculty of Medical Sciences, Department of Cardiovascular Medicine, Kyushu University, Fukuoka, Japan

**Keywords:** coronary vasospasm, intercoronary communication, myocardial stunning

## Abstract

**Background:**

Intercoronary communication (ICC) is an extremely rare congenital coronary anomaly that is generally considered benign. We herein report a novel case in which an ICC itself caused coronary vasospasm, leading to myocardial ischemia.

**Case Summary:**

A 79-year-old woman presented with rest angina. Transthoracic echocardiography revealed hypokinesia of the posterolateral left ventricular wall. Coronary angiography demonstrated an ICC between the left circumflex artery and right coronary artery, and acetylcholine-induced focal coronary vasospasm was provoked exclusively in the ICC and was relieved with intracoronary isosorbide dinitrate. The patient was treated with oral vasodilators, after which she remained symptom-free. Follow-up echocardiography showed complete recovery of wall motion.

**Discussion:**

This case illustrates a paradoxical scenario in which an ICC, typically considered as a “safety valve,” instead caused localized vasospastic myocardial ischemia.

**Take-Home Messages:**

ICC itself may cause coronary vasospasm, resulting in myocardial ischemia.

## Visual Summary

**Figure:**
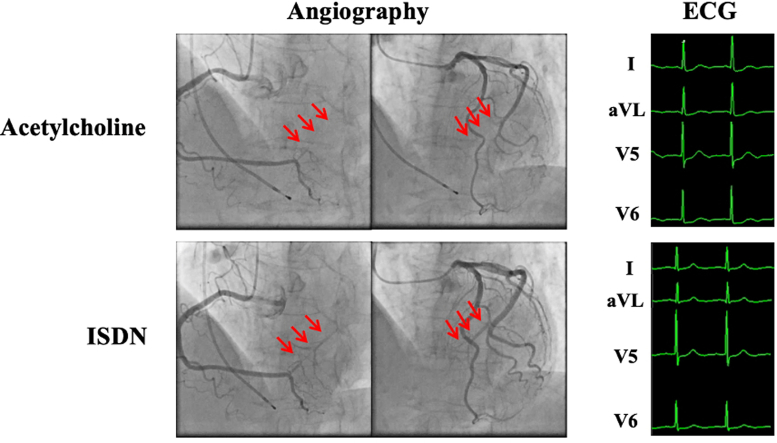

## History of Presentation

A 79-year-old Japanese woman with no history of hypertension, hyperlipidemia, diabetes mellitus, or smoking presented with new-onset chest pain at rest. She had experienced several episodes of pressure-like chest pain occurring in the early morning hours and at rest, each lasting a few minutes and resolving spontaneously. There was no history of exertional angina. Vital signs were within normal limits, and physical examination was unremarkable.

**Take-Home Messages**

• ICC itself may cause vasospasm and lead to myocardial ischemia.
• Clinicians should consider provocative spasm testing in patients with angina and angiographically normal coronaries, especially if congenital anomalies such as an ICC are present.

## Past Medical History

The patient had no medical history of hypertension, hyperlipidemia, or diabetes mellitus.

## Differential Diagnosis

Although vasospastic angina was a leading concern, the differential diagnosis included microvascular angina and stress-induced cardiomyopathy.

## Investigations

**Figure 1:**
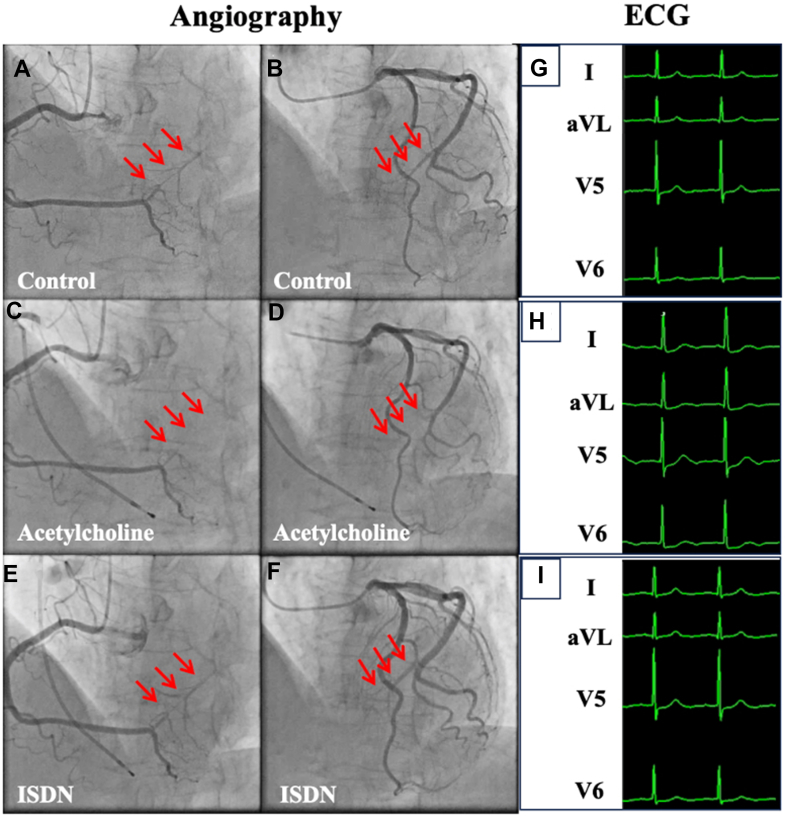
Coronary Angiography, Acetylcholine Provocation Testing, and Electrocardiogram Control angiography demonstrated an intercoronary communication (red arrows) between the right coronary artery and the left circumflex artery (A and B), which became narrowed and partially invisible after acetylcholine infusion (C and D). The vasospasm was subsequently relieved after administration of isosorbide dinitrate (E and F). During acetylcholine infusion, ischemic changes on ECG were observed, including ST-segment depression in leads I and aVL, and further ST-segment depression in leads V_5_ and V_6_ (G to I). ECG = electrocardiogram; ISDN = Isosorbide dinitrate.

Electrocardiogram showed normal sinus rhythm without ischemic changes. Cardiac biomarkers (troponin-T and creatine kinase-MB) were within normal limits. Transthoracic echocardiography revealed a mild hypokinesis of the posterolateral wall of the left ventricle, with overall preserved ejection fraction. In coronary angiography, the left and right coronary arteries were selectively engaged and injected separately, following routine practice. There were no significant atherosclerotic stenoses in the major coronary arteries. However, an unusual finding was observed: a direct arterial connection between the distal left circumflex artery (LCx) and the right coronary artery (RCA) in the atrioventricular groove. This intercoronary communication (ICC) was a large-caliber channel (approximately 1.5 mm in diameter) and exhibited competitive bidirectional flow (Figures 1A and 1B, Videos 1 and 2). The ICC provided dual perfusion to the posterior-lateral myocardial region, corresponding to the area of wall motion abnormality on echocardiogram. Intracoronary acetylcholine (ACh) provocation testing was performed to evaluate vasospasm. ACh was administered at a dose of 100 μg into the left coronary artery and 50 μg into the RCA. Notably, during ACh infusion, a focal epicardial vasospasm was observed exclusively in the ICC segment, whereas the native RCA and LCx showed no evidence of epicardial spasm. Angiographically, the ICC constricted severely, and its previously brisk flow was completely interrupted during the spasm (Figures 1C and 1D, Videos 3 and 4). At that moment, the patient reported typical chest and neck pain, accompanied by ischemic changes on electrocardiogram, including ST-segment depression in leads I and aVL, and further ST-segment depression in leads V_5_ and V_6_ (Figures 1G to 1I). Intracoronary isosorbide dinitrate was given, resulting in prompt resolution of the ICC spasm and restoration of normal flow through the channel (Figures 1E and 1F, Videos 5 and 6). The patient's chest pain abated almost immediately, and no arrhythmias or complications occurred.

These findings indicated that the symptoms were due to episodic spasm of the ICC, and the persistent wall motion abnormality represented myocardial stunning from repeated ischemia in that territory.

## Management

The patient was started on optimal medical therapy for vasospastic angina. Calcium channel blockers (nifedipine 40 mg daily) and long-acting nitrates were prescribed, along with sublingual nitroglycerin as needed. She was advised to avoid smoking and extreme cold exposure, and to minimize stress, all of which can precipitate coronary spasm.

## Outcome and Follow-Up

Over the following months, the patient reported complete resolution of angina symptoms on the prescribed regimen. A repeat echocardiogram after 3 months of therapy showed normalization of left ventricular wall motion in the posterolateral region. This recovery confirmed myocardial viability and was consistent with reversible myocardial stunning. The patient continues to do well on follow-up with no evidence of recurrent angina.

## Discussion

ICC is a rare congenital coronary anomaly between ≥2 coronary arteries, allowing bidirectional or unidirectional blood flow between them.^1^ They are distinct from acquired coronary collaterals in both anatomy and histology. Angiographically, collateral vessels in chronic occlusive disease are usually small (<1 mm), tortuous, or helical channels, whereas ICCs tend to be larger (≥1 mm in diameter), fairly straight, and run on the epicardial surface.^2^ These features reflect the congenital nature of ICCs, possibly representing persistence of embryonic coronary plexus connections.^3^ ICCs are exceedingly rare, with angiographic prevalence reported between about 0.002% to 0.05%.^2^^,^^4^ Two major patterns of ICC have been described: one between the distal left anterior descending and posterior descending artery in the ventricular apex, and another between the distal RCA and LCx in the atrioventricular groove,^1^ as observed in this case.

An ICC can function as a natural bypass. Previous reports have shown that if one artery develops spasm or occlusion, the ICC may supply the jeopardized myocardial territory from the other contralateral artery, thereby preventing ischemia.^3^^,^^5^ Indeed, some authors have described ICCs as a “protective coronary arcade” or a hemodynamic “safety valve” for the myocardium.^5^^,^^6^ However, ICCs are not universally benign. Unidirectional intercoronary connections have been associated with a potential coronary steal phenomenon, in which blood flow is diverted away from ischemic regions.^1^ Rare cases of unidirectional RCA-LCx communications have been reported as a possible cause of angina, potentially due to such steal physiology.^7^^,^^8^ To our knowledge, epicardial vasospasm occurring within an ICC has not been reported previously. In our patient, however, ICC itself could cause coronary vasospasm, resulting in myocardial ischemia. These findings suggest that the net hemodynamic impact of an ICC may be context dependent.

Several plausible mechanisms may explain the hyperreactivity of the ICC to vasoconstrictive stimuli. These include structural or developmental anomalies, and endothelial dysfunction secondary to disturbed shear stress. Unlike thin-walled collaterals, ICCs usually possess a fully developed smooth muscle layer and are thus capable of substantial contraction.^3^ In addition, the hemodynamics within an ICC are unusual. In cases with bidirectional flow, shear stress may be oscillatory or reduced, which can impair endothelial function. We speculate that such competing flow in the ICC may hamper shear-mediated endothelial nitric oxide synthase activation. A consequent reduction in basal nitric oxide production could predispose the vessel to vasospasm because ACh and other provocative agents tend to induce vasoconstriction when endothelial nitric oxide bioavailability is diminished.^9^

Once vasospastic angina is diagnosed, aggressive medical management with vasodilators is warranted. In our patient, dramatic clinical improvement and the resolution of wall motion abnormalities after 3 months of calcium - channel blocker and nitrate therapy underscore the effectiveness of targeting coronary vasospasm. Stunning myocardium is typically described after transient episodes of acute ischemia followed by reperfusion, where contractile dysfunction persists despite restoration of blood flow. In the present case, however, intermittent ICC spasm appeared sufficient to induce chronic repetitive ischemia leading to reversible myocardial stunning, a condition recognized as “vasospastic angina-induced cardiomyopathy.”^10^

## Conclusions

This case demonstrates a rare mechanism of myocardial ischemia caused by focal vasospasm within an ICC, a structure typically considered hemodynamically benign. It emphasizes the importance of recognizing that even congenital coronary anomalies can become pathologic under certain conditions. Careful provocation testing enabled precise localization of the spasm, allowing for effective targeted therapy. Prompt recognition and optimized vasodilator treatment can lead to full recovery of cardiac function, even in cases of chronic myocardial stunning.

**Visual Summary:**
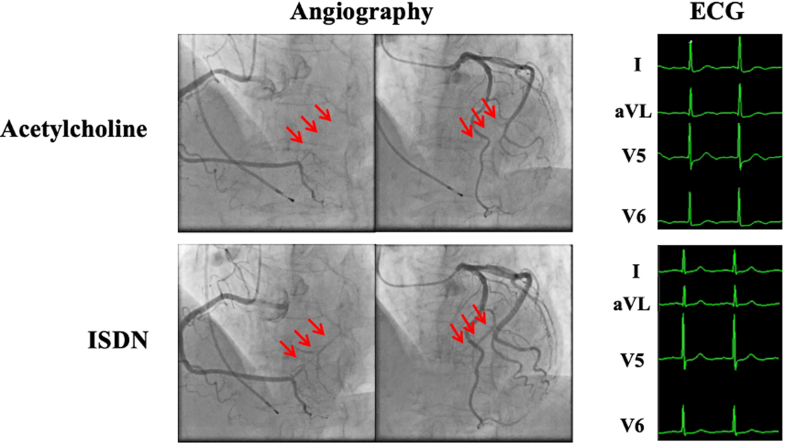
Acetylcholine-Induced Vasospasm Within an Intercoronary Communication (Left panels) Angiography showing an intercoronary communication and acetylcholine-induced focal vasospasm (red arrows) relieved by isosorbide dinitrate. (Right panels) ECG showing transient ST-segment depression during spasm with complete resolution after isosorbide dinitrate administration. ECG = electrocardiogram; ISDN = Isosorbide dinitrate.

## Funding Support and Author Disclosures

The authors have reported that they have no relationships relevant to the contents of this paper to disclose.

## Appendix

Video 1 Left Coronary Angiography (Baseline) Showing Intercoronary Communication Without Vasospasm

Video 2 Right Coronary Angiography (Baseline) Showing Intercoronary Communication Without Vasospasm

Video 3 Left Coronary Angiography During Acetylcholine Provocation Demonstrating Vasospasm Within the Intercoronary Communication

Video 4 Right Coronary Angiography During Acetylcholine Provocation Showing Dynamic Narrowing of the Intercoronary Communication

Video 5 Left Coronary Angiography After Intracoronary Isosorbide Dinitrate Showing Resolution of Vasospasm

Video 6 Right Coronary Angiography After Intracoronary Isosorbide Dinitrate Showing Restoration of the Intercoronary Communication Caliber
